# Trends and patterns of benzodiazepines and Z‐drugs prescriptions in Australian general practice: A national study (2011–2018)

**DOI:** 10.1111/dar.13561

**Published:** 2022-10-10

**Authors:** David Gonzalez‐Chica, Mumtaz Begum, Carla Bernardo, Elizabeth Hoon, Alexander Sweetman, Nigel Stocks

**Affiliations:** ^1^ Discipline of General Practice Adelaide Medical School, Faculty of Health and Medical Sciences, The University of Adelaide Australia; ^2^ Adelaide Institute for Sleep Health and Flinders Health and Medical Research Institute, Sleep Health, Flinders University Australia; ^3^ Adelaide Rural Clinical School, Faculty of Health and Medical Sciences The University of Adelaide Australia

**Keywords:** anxiolytics, benzodiazepines, electronic health records, general practice, hypnotics

## Abstract

**Introduction:**

We aimed to explore trends and sociodemographic patterns in benzodiazepine (BZD) (by half‐life) and Z‐drugs prescribing in Australian general practice.

**Methods:**

This open cohort study used de‐identified electronic health records of 1.4 million patients (50,812,413 consultations) from 402 Australian practices (MedicineInsight 2011–2018). Annual prescribing frequency and changes over time were estimated according to sex, age, socioeconomic position and rurality.

**Results:**

Between 2011 and 2018, the prescribing of very short‐acting BZD increased from 0.10 to 0.29 per 1000 consultations (average annual change +17.2% [95% CI 9.6; 25.3]), while it declined for short‐intermediate (from 38.5 to 26.6 per 1000 consultations; annual change −5.1% [95% CI −5.6; −4.5]), long‐acting BZD (from 24.1 to 21.6 per 1000 consultation; annual change −1.5% [95% CI −2.2; −0.8]) and Z‐drugs (from 4.6 to 4.0 per 1000 consultations; annual change −1.9% [95% CI −3.0; −0.7]). Short‐intermediate‐acting BZD prescribing was three times more frequent among women aged 65+ years than younger women, and long‐acting BZD three‐to‐four times more likely among younger than older men. Z‐drugs prescribing was higher among women aged 45–64 years than younger or older females. Short‐intermediate‐ and long‐acting BZD were more likely prescribed for patients from more disadvantaged areas, and Z‐drugs in more advantaged areas. There were no disparities by rurality.

**Discussion and Conclusions:**

Although most BZD and Z‐drugs prescriptions declined over time, short‐intermediate BZD prescriptions remained higher among older women and long‐acting BZD more frequent among younger men, especially for those living in more disadvantaged areas. Targeted interventions could reduce the prescribing of BZD and Z‐drugs in these groups.

## 1 INTRODUCTION

Benzodiazepines (BZD) and Z‐drugs are medications with calming and sedative effects commonly used to treat anxiety and insomnia. These medications are recommended for short‐term (approximately 4 weeks) management and only when first‐line therapy (e.g., cognitive behavioural therapy) is unsuccessful [1]. However, when used for long‐term management, these drugs increase the risk of dependency, impaired cognitive functioning, dementia, falls/fractures, accidents, hospitalisations and overdose/accident‐related mortality [1, 2, 3]. In Australia, the number of deaths involving BZD rose by 58% between 2011 and 2018 (from 2.4 to 3.8 per 100,00 population), with a higher increase in metropolitan areas [4]. Despite these risks, the prevalence of BZD prescribing is high, especially among older adults, women and socioeconomically disadvantaged populations in the US, Canada, Spain, New Zealand and Australia [5, 6, 7, 8, 9, 10, 11].

BZD can be classified depending on different characteristics (e.g., main use, speed of onset, elimination half‐life). According to their elimination half‐lives, BZD are usually classified as: (i) short‐intermediate‐acting BZD (half‐life ≤24 h), usually indicated for the management of anxiety disorders, panic attacks, refractory phobias and insomnia; or (ii) long‐acting BZD (half‐life >24 h), usually indicated as anxiolytics, anticonvulsants, sedatives in palliative care and/or for insomnia management [6, 12, 13]. On the other hand, Z‐drugs (e.g., zolpidem and zoplicone) are used for managing insomnia due to their short half‐life (1–6 h) and fewer adverse effects compared to BZD [1, 14, 15]. Despite these differences, Z‐drugs carry similar risks of misuse, dependence and falls/injuries as BZD [13, 16, 17].

Nonetheless, few studies have reported patterns of BZD prescribing according to the elimination half‐life. Investigating these prescribing patterns is relevant, considering short‐intermediate‐acting BZD are associated with a greater risk of rebound or withdrawal symptoms than long‐acting BZD [1, 13, 18]. According to the available literature, short‐intermediate‐acting BZD are more prescribed than long‐acting BZD in Spain [5] (prescription billing data), Ireland [19] (pharmacy claims database) and the USA [12] (National Ambulatory Medical Care Survey). However, the evidence about changes over time in the prescribing of short‐intermediate‐ and long‐acting BZD is inconsistent across countries [5, 6, 12].

In Australia, most previous studies on BZD and Z‐drugs prescriptions have included either a small sample, only older people or did not distinguish prescribing patterns by BZD half‐life [8, 9, 20, 21]. Many of these studies used dispensing data, which lacks information on sociodemographic characteristics. Miller and colleagues used the Bettering the Evaluation And Care of Health study data to investigate patterns of medicine prescribing for insomnia, including BZD and Z‐drugs, in Australian primary care between 2000 and 2015 [11]. It was reported that 90% of encounters for management of insomnia resulted in a medicine prescription, with some evidence of decreasing temazepam and increasing zopiclone prescribing over time. However, associations between prescribing behaviours and sociodemographic information were not reported.

Therefore, this study aimed to elucidate the sociodemographic patterns in prescribing BZD by half‐life and Z‐drugs, and the changes in prescribing over time, using a large Australian nationwide primary care database with more than 50 million consultations between 2011 and 2018.

## 2 METHOD

### 2.1 Data source

In this open cohort study, we used data from a national Australian primary care database called MedicineInsight, established in 2011. The database consists of de‐identified electronic health records from approximately 662 general practices (8.2% of all Australian general practices) and over 2700 general practitioners across Australia [22]. MedicineInsight contains information on patient demographic characteristics, diagnoses, laboratory results and medications prescribed, which are routinely recorded by general practitioners using standard medical terminologies. Most Australian general practices use the coding systems ‘Docle’, ‘Pyefinch’ or the International Classification of Primary Care 2. Each patient and practice is assigned a unique ID, which is used for follow‐up of the same patient over time, and for merging different subsets of data during analyses.

### 2.2 Study population

All methods used for selecting participants, data extraction and analyses followed recommendations from the REporting of studies Conducted using Observational Routinely‐collected health Data (RECORD) Statement [23]. Further details on the methods have been described elsewhere [22, 24]. Only data from practices with consistent data provision were included (i.e., no gap of more than 6 weeks in data provision in the previous 2 years and a ratio between the highest and lowest number of annual consultations [2011–2018] lower than five). The study included all regular patients (i.e., at least three visits in any two consecutive years [e.g., a regular patient in 2018 had at least 3 clinical encounters from 1 January 2017 to 31 December 2018 for calendar year 2018], with at least one visit in each of these two consecutive years) aged 18+ years who attended general practices participating in MedicineInsight from 1 January 2011 to 31 December 2018 [22]. All clinical encounters of these regular patients were included (i.e., any visit where a diagnosis, reason for encounter or script was recorded in the electronic health records), but administrative contacts (e.g., phone calls, reminders) were excluded. To minimise potential duplicated encounters, we limited the daily clinical encounters count to one encounter per patient. The final sample consisted of 1,450,613 adult patients, accounting 50,812,413 consultations from 404 general practices (Table S1, Supporting Information).

### 2.3 Data extraction

Data on BZD and Z‐drugs were extracted from the ‘script item’ dataset using active ingredients and commercial brand names of drugs approved in Australia [1, 14]. The name and elimination half‐life of each BZD and Z‐drugs are provided in Table 1. Although BZD are usually classified as short‐intermediate‐ or long‐acting BZD, for the purposes of this study, midazolam and triazolam were classified separately as very short‐acting BZD, as they have a shorter half‐life (1–4 h) and different use (i.e., sedatives for short medical procedures, to induce anaesthesia and for short management of anxiety/insomnia) compared to other short‐intermediate‐acting BZD [1, 12, 14]. Therefore, the investigated drugs were classified as very short‐, short‐intermediate‐ or long‐acting BZD, or Z‐drugs.

**TABLE 1:** Pharmacokinetic classification, elimination half‐life and total prescriptions of benzodiazepines and Z‐drugs between 2011 and 2018

	Elimination half‐life, hours	Total prescriptions
Very short‐acting		
Midazolam	1.4–2.4	4534
Triazolam	1–3	1613
Short‐intermediate‐acting		
Alprazolam	6–25	99,272
Bromazepam	20	8638
Lorazepam	12–16	44,838
Oxazepam	4–15	452,004
Temazepam	5–15	999,785
Long‐acting		
Clobazam	17–49	4975
Clonazepam	22–54	45,184
Diazepam	20–80	991,441
Flunitrazepam	20–30	10,221
Nitrazepam	16–48	155,188
Z‐drugs		
Zolpidem	2.5	132,740
Zopiclone	5	78,063

### 2.4 Outcome

The prescribing frequency (per 1000 consultations) of BZD and Z‐drugs (i.e., prescribed yes or no) and the average annual percent change in the prescribing of every very short‐acting (≤3 h), short‐intermediate‐acting (4–24 h), long‐acting BZD (>24 h) or Z‐drugs between 2011 and 2018 were considered the outcomes of the study.

### 2.5 Sociodemographic variables (covariates)

Sociodemographic characteristics included sex (male, female), age groups (18–44 years, 45–64 years, ≥65 years), the remoteness of the practice (major cities, inner regional, outer regional/remote/very remote) and two macro‐economic indicators of socioeconomic position (the Index of Relative Socioeconomic Advantage and Disadvantage [IRSAD] based on the patient's residence and the IRSAD based on the practice location). The IRSAD is an area‐level measure of socioeconomic advantage and disadvantage developed by the Australian Bureau of Statistics using 2016 census data on education, employment/income and housing, and ranks areas from most disadvantaged to most advantaged [25]. The corresponding IRSAD score for the patients and the practice was generated by MedicineInsight based on the corresponding patient residential or practice location postcode. Both patient and practice IRSAD quintiles were generated and used in the analyses.

### 2.6 Statistical analysis

All analyses were conducted on Stata/MP15.1 (StataCorp, Texas, USA), considering general practices as clusters and using robust standard errors. Annual prescription frequencies (per 1000 consultations) for each year between 2011 and 2018 were estimated by dividing the number of consultations with a BZD or Z‐drug prescription as the numerator and the number of total consultations as the denominator (i.e., binary outcome). The association between sociodemographic variables and the prescribing of BZD or Z‐drugs was explored using logistic regression, considering two models for adjustment. Initially, only practice remoteness and IRSAD (i.e., practice characteristics) were included in the logistic regression analyses (Model 1), with the adjusted results for these two variables reported in figures/tables. Subsequently, patient‐level characteristics (age, sex and patient IRSAD) were also included in the regression analyses (Model 2) to obtain adjusted results for these three variables (e.g., age was adjusted for sex, patient IRSAD, practice remoteness and IRSAD). The *p*‐values for the association between each sociodemographic variable and the prescription status of each drug type were obtained using Wald tests for heterogeneity instead of likelihood‐ratio tests due to the clustering design. To test the possible interaction between age and sex, an additional model including a multiplicative term between these two variables was used. A *p*‐value lower than 0.01 was considered evidence of an interaction. Finally, to facilitate interpretability, all results from the logistic regression models were presented as predicted adjusted probabilities (i.e., frequencies) instead of odds ratios using the Stata command ‘margins’. That command calculates adjusted outcome probabilities for each category of the independent variable, averaging or integrating the results over the remaining covariates included in the regression model (i.e., confounders). All these adjusted results were presented graphically (adjusted frequencies per 1000 consultations) and as supplementary tables.

Poisson regression was used to estimate the average percent annual change in the prescribing of the investigated drugs by sociodemographic characteristics between 2011 and 2018. The same approach used for adjustment using logistic regression was applied in this case. In addition, we included a multiplicative term between each investigated covariate and the year (as an ordinal variable) to test differences in the observed trends according to sociodemographic variables. Wald tests for trend were used to obtain the corresponding *p*‐values. Finally, to obtain the average percent annual change in the prescribing of the investigated drugs, rate ratios from these multiplicative terms were converted to a percent using the formula (rate ratio – 1) × 100.

All analyses were repeated considering the patient as the unit for analysis (i.e., proportion of patients prescribed BZD or Z‐drugs) and presented as sensitivity analysis.

### 2.7 Ethical considerations

The independent MedicineInsight Data Governance Committee approved the study (protocol 2019–029). The Human Research Ethics Committee of the University of Adelaide exempted the study from the ethical review due to the use of non‐identifiable data.

## 3 RESULTS

A total of 1,450,613 regular adult patients contributed to 50,812,413 consultations recorded between 2011 and 2018. Of the total consultations, 61% were recorded for female patients and 44.6% for individuals aged 65+ years (22.7% for 18–44 years and 32.7% for 45–64 years old). Overall, there was a total of 2,844,071 clinical encounters where BZD or Z‐drugs were prescribed (5.6% of all consultations recorded). Of these, 0.2% were very short‐acting, 51.7% were short‐intermediate‐acting, 40.7% long‐acting BZD, and 7.4% were Z‐drugs.

### 3.1 Very short‐acting BZD

**FIGURE 1:**
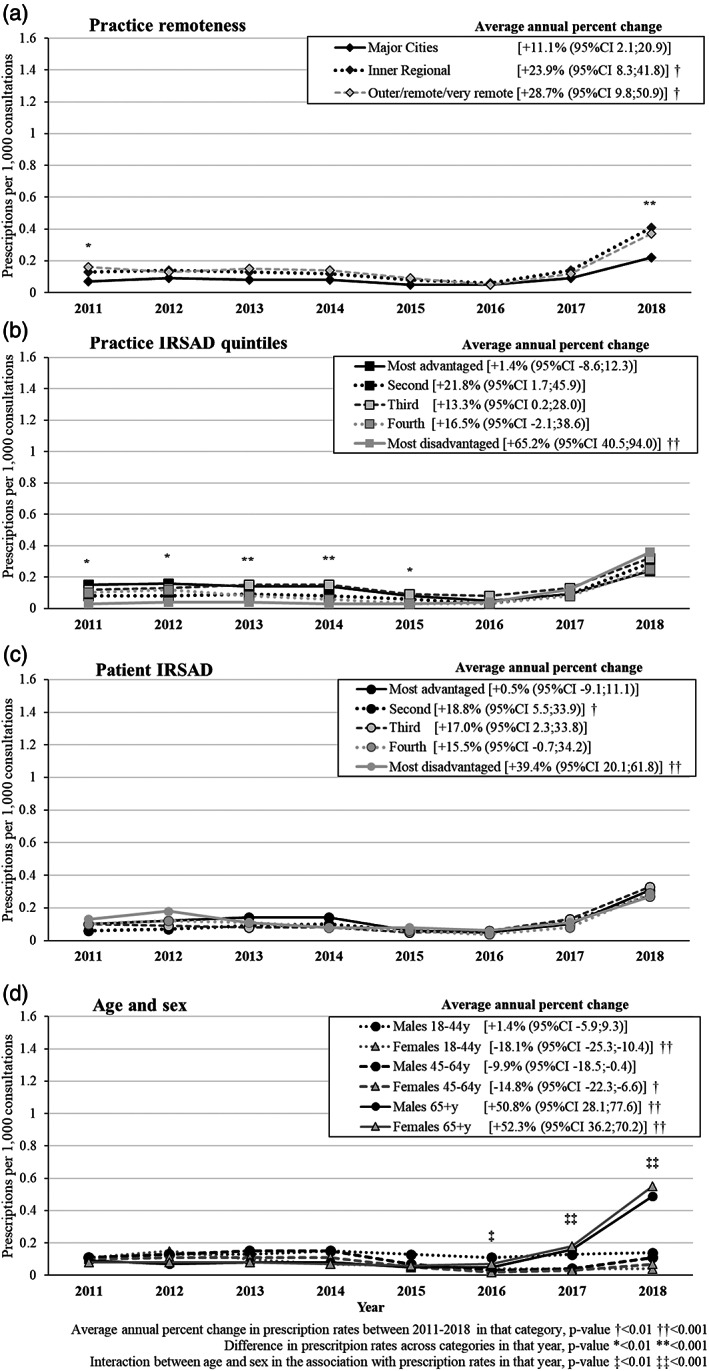
Adjusted results about the prescription of very short‐acting benzodiazepines per 1000 consultations by sociodemographic characteristics and average annual percent change from 2011 to 2018. Marginal adjusted frequencies based on logistic regression models. Practice remoteness and practice index of relative socioeconomic advantage and disadvantage (IRSAD) were mutually adjusted (Model 1). Patient age, gender and IRSAD were mutually adjusted and also for practice remoteness and practice IRSAD (Model 2)

Despite the low number of prescriptions of very short‐acting BZD compared to any other group, between 2011 and 2017, prescribing frequencies for this type of medication ranged between 0.05 in 2011 and 0.11 per 1000 consultations, increasing to 0.29 per 1000 consultations in 2018 (average annual change +17.2% [95% confidence interval; CI 9.6, 25.3]; Tables S2 and S3, Supporting Information). Figure 1 shows that the prescribing pattern of very short‐acting BZD was similar among practices in major cities, inner regional or outer/remote/very remote settings. However, the prescribing frequency observed in 2018 was two times higher in outer/remote/very remote practices (0.41 per 1000 consultations in inner regional areas) compared to major cities (0.22 per 1000 consultations; *p*‐value <0.001). Very short‐acting BZD were 3–5 times more prescribed among practices located in higher IRSAD (most advantaged) areas compared to most disadvantaged settings until 2015 (*p*‐value <0.01 in any year between 2011 and 2015), when these differences became less evident. For instance, in 2016, the prescribing frequency was 0.05 per 1000 consultations in most advantaged areas versus 0.04 in most disadvantaged areas. The patient IRSAD was not associated with the prescription frequencies recorded in any year. Until 2016, the recorded prescribing of very short‐acting BZD was similar for males and females of any age. Then, a rapid increase in the prescribing of very short‐acting BZD was observed among males and females aged 65+ years, increasing from 0.06 per 1000 consultations in 2016 to 0.52 per 1000 consultations in 2018 (*p*‐value for interaction between age and sex <0.01).

### 3.2 Short‐intermediate‐acting BZD

**FIGURE 2:**
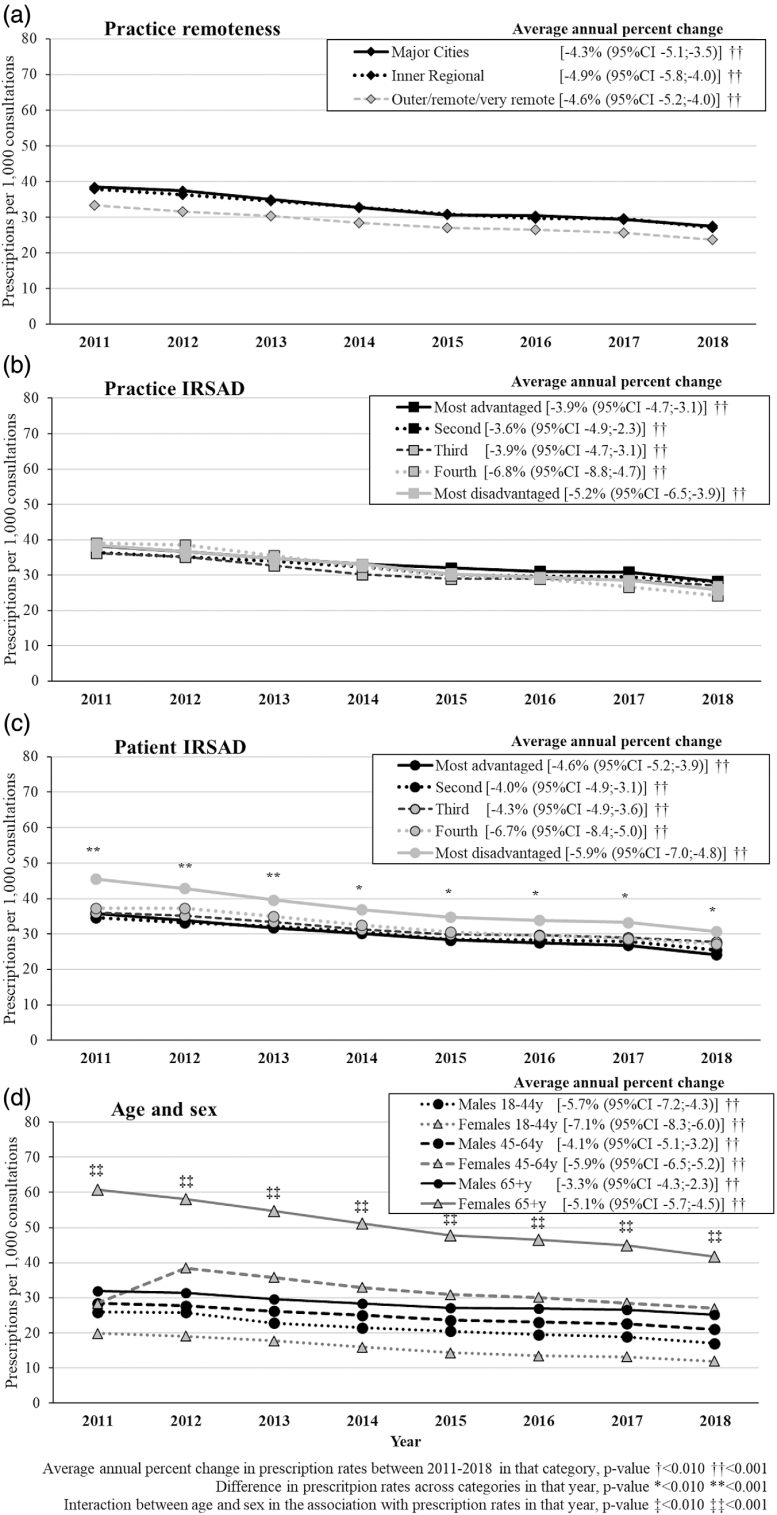
Adjusted results about the prescription of short‐intermediate‐acting benzodiazepines per 1000 consultations by sociodemographic characteristics and average annual percent change from 2011 to 2018. Marginal adjusted frequencies based on logistic regression models. Practice remoteness and practice index of relative socioeconomic advantage and disadvantage (IRSAD) were mutually adjusted (Model 1). Patient age, gender and IRSAD were mutually adjusted and also for practice remoteness and practice IRSAD (Model 2).

The recorded prescription of short‐intermediate‐acting BZD decreased from 38.5 in 2011 to 26.6 per 1000 consultations in 2018 (average annual change −5.1% [95% CI −5.6,−4.5]; Tables S4 and S5, Supporting Information). Figure 2 shows this pattern was independent of practice rurality or IRSAD. However, these prescriptions were more likely to be provided to patients living in areas with a lower IRSAD (most disadvantaged areas) than those living in more advantaged areas (*p*‐value <0.01 in any year). The prescription of short‐intermediate‐acting BZD was three times more likely to be recorded among women aged 65+ years than women in the 18–44 age group in any year (*p*‐value for interaction between age and sex <0.001 in any year). Overall, differences in prescription frequencies and changes over time according to age were less apparent among males.

### 3.3 Long‐acting BZD

**FIGURE 3:**
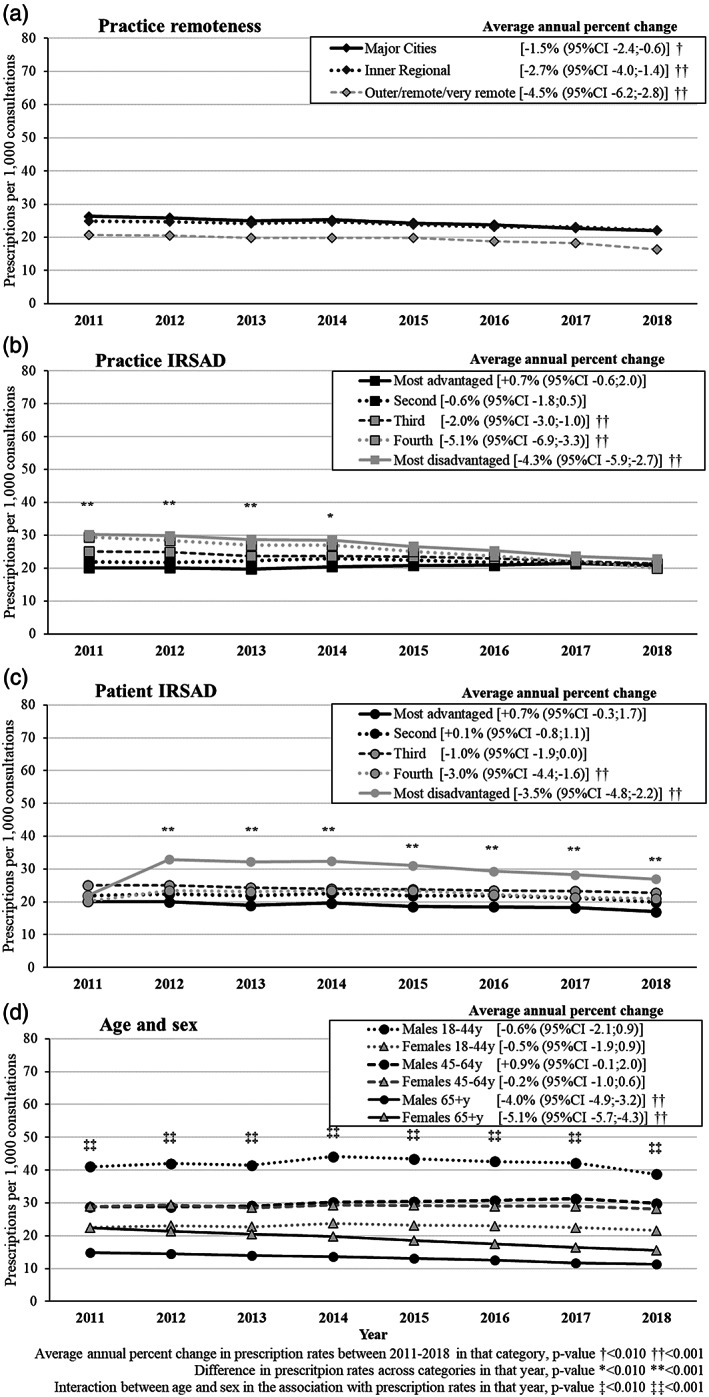
Adjusted results about the prescription of long‐acting benzodiazepines per 1000 consultations by sociodemographic characteristics and average annual percent change from 2011 to 2018. Marginal adjusted frequencies based on logistic regression models. Practice remoteness and practice index of relative socioeconomic advantage and disadvantage (IRSAD) were mutually adjusted (Model 1). Patient age, gender and IRSAD were mutually adjusted and also for practice remoteness and practice IRSAD (Model 2).

The reduction over time in the recorded prescription of long‐acting was less marked (average annual change −1.5% [95% CI −2.2, −0.8]; Tables S6 and S7, Supporting Information) than for short‐intermediate‐acting BZD. Figure 3 shows no differences in the prescription of these drugs among practices located in major cities, inner regional or outer/remote/very remote settings, and differences according to practice IRSAD disappeared over time (*p*‐value <0.01 between 2011 and 2014 only). Except for 2011, long‐acting BZD prescriptions were 55–70% more likely to be recorded among individuals living in the most disadvantaged areas than those in the top IRSAD quintile (most advantaged areas; *p*‐value <0.001 in any year between 2012 and 2018). Prescription frequencies for males and females aged 65+ years decreased 4–5% per year (Table S7, Supporting Information and Figure 3), but remained steady in the other age groups. There was a two‐way interaction between sex and age on long‐acting BZD prescriptions for each year (*p*‐value for interaction <0.001 in any year). Overall, the recorded prescription of long‐acting BZD was 3–4 times higher among males aged 18–44 years than males aged 65+ years. Among women, age differences in prescription frequencies were less pronounced.

### 3.4 Z‐drugs

**FIGURE 4:**
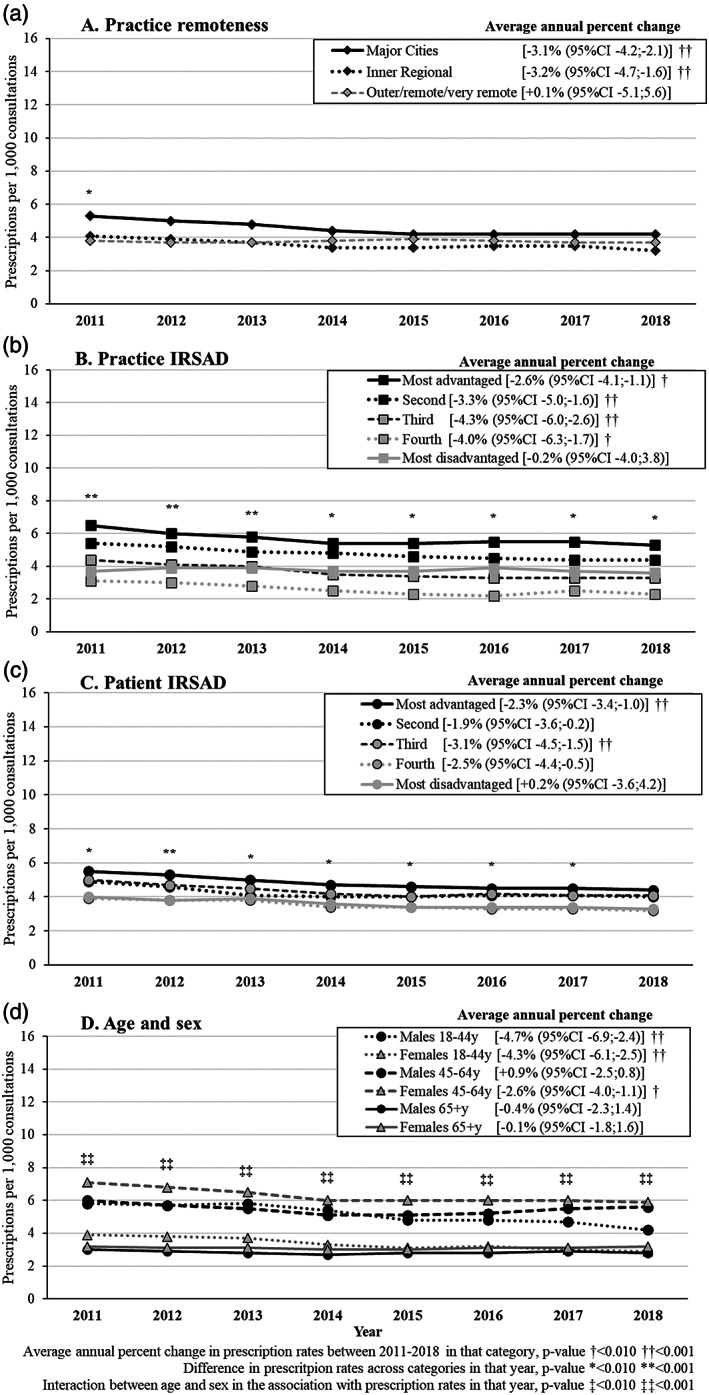
Adjusted results about the prescription of Z‐drugs per 1000 consultations by sociodemographic characteristics and average annual percent change from 2011 to 2018. Marginal adjusted frequencies based on logistic regression models. Practice remoteness and practice index of relative socioeconomic advantage and disadvantage (IRSAD) were mutually adjusted (Model 1). Patient age, gender and IRSAD were mutually adjusted and also for practice remoteness and practice IRSAD (Model 2).

The recorded prescription of Z‐drugs diminished on average 1.9% per year (95% CI −3.0, −0.7), decreasing from 4.6 in 2011 to 4.0 per 1000 consultations after 2013 (Tables S8 and S9, Supporting Information). Figure 4 shows Z‐drugs prescriptions were largely similar in major cities, inner regional or outer/remote/very remote settings (ranging from 3.7 to 3.9 per 1000 consultations in outer regional/remote/very remote areas and 4.2 to 5.0 per 1000 consultations in major cities from 2012 to 2018). Nonetheless, the recorded prescription of Z‐drugs was twice as high for practices with a higher IRSAD (most advantaged) than those in the most disadvantaged areas (lower IRSAD; *p*‐value <0.01 in any year). A similar pattern was observed according to the patient's IRSAD, but the disparities were less evident. Z‐drugs prescriptions were more likely to be recorded among males or females aged 45–64 years, and the lowest frequency was observed among those aged 65+ years (*p*‐value for interaction between age and sex <0.001 in any year).

### 3.5 Sensitivity analyses

Tables S10 to S13, Supporting Information, present the proportion of patients prescribed BZD or Z‐drugs between 2011 and 2018. The observed associations were similar to the consultation‐level results presented above.

## 4 DISCUSSION

This study used a large national primary care database to explore trends and sociodemographic patterns in the prescribing of BZD according to their half‐life and Z‐drugs between 2011 and 2018. Very short‐acting BZD were the only medications with a rise in prescribing in recent years (17% over the study period). This upsurge was related to an increase in the prescription of these drugs among patients aged 65+ years and in practices located in rural settings, which probably reflects their use as sedatives for short medical procedures in rural areas [1, 14]. However, the total number of those prescriptions was very low compared to the other medication groups.

Overall, 40.7% of the prescribed BZD or Z‐drugs were for long‐acting BZD, which is higher than findings in Spain [5], Canada [15] and the USA [6], where only one‐quarter of these prescriptions were for long‐acting BZD. Moreover, according to our findings, short‐intermediate, long‐acting BZD and Z‐drugs showed a similar reduction pattern over time. In the USA, both short‐intermediate‐ and long‐acting BZD prescriptions increased between 2003 and 2015 [12, 26]. In Spain, a rise in short‐intermediate‐acting BZD and a decline in long‐acting BZD and Z‐drugs prescribing was observed between 2002 and 2015 [5], while a reduction in the prescription of these drugs was reported in Ireland (2005–2015) [19] and Denmark (2003–2013) [27].

According to the available literature, findings on the prescription of short‐intermediate‐ and long‐acting BZD by sex are also mixed, with a higher proportion of long‐acting BZD prescribing among men than women in the USA (2008) [6] and an increase in short‐intermediate‐acting BZD prescription among women in Spain (2002–2015) [5]. In our study, the prescribing of short‐intermediate‐acting BZD was 3–4 times more likely to be recorded for older women (i.e., they were prescribed in 4 out of 100 consultations among females aged 65+ years versus in 1 out of 100 consultations among younger women), and long‐acting BZD were four times more frequent among younger men (i.e., they were prescribed in 4 out of 100 consultations among males aged 18–44 years vs. in 1 out of 100 consultations among older men). Although inter‐country differences could result from sample characteristics (patient vs. general population), study period and definitions used, they do not explain the higher proportion of long‐acting BZD compared to the other drugs or the stagnation of prescription frequencies in young and middle‐aged adults over time.

The higher prescription of long‐acting BZD among younger males (18–44 years old in our study) may be because men are less likely to seek help from a psychologist for managing anxiety or insomnia [28]. Nonetheless, BZD prescribing to young people might have a higher potential for misuse. In Australia, people aged 20–39 years had the highest level of non‐medical use of BZD in 2016, while males aged 30–49 years had the highest number of drug‐induced deaths involving BZD between 2013 and 2017 [4, 29]. Similarly, males accounted for two‐thirds of drug‐induced deaths involving BZD (1630 deaths among males vs. 836 in females) [29].

Short‐intermediate‐ and long‐acting BZD were also more prescribed among patients living in the most disadvantaged areas, which is consistent with studies from France and Canada, where people with lower education, lower occupational grades, unemployed or lower income were more likely to be prescribed BZD [10, 30]. Previous studies have shown that people from disadvantaged neighbourhoods have a higher prevalence of psychological distress [31, 32], face more barriers when they try to access non‐pharmacological treatments [33], and are more likely to receive medications for anxiety and depression than those from more advantaged settings [34].

On the other hand, the prescribing of Z‐drugs was more closely related to the practice than the individual socioeconomic position, as the prescribing of these drugs was twice as likely to be recorded in practices located in more advantaged than disadvantaged areas. Z‐drugs were also more prescribed for middle‐aged patients than in the elderly. These findings could be explained by the fact that zolpidem can only be obtained as a private prescription as it is not subsided by the government's Pharmaceutical Benefits Scheme [1, 14, 35].

Cognitive behavioural therapy and other non‐pharmacological options such as brief behavioural therapy and stepped care models are effective long‐term and recommended first‐line treatment for insomnia, anxiety and comorbid depressive symptoms [33, 36, 37]. However, less than 1% of primary care patients in Australia currently access these treatments [11, 33]. Therefore, the high prevalence of BZD and Z‐drugs prescribing in Australian general practice is probably influenced by the unavailability of accessible referral pathways to these and other specialised services (e.g., BZD withdrawal support), the lack of clarity about funding for non‐pharmacological management, and poor financial reimbursement for the general practitioners when managing these conditions (i.e., current funding structure favours short consultations and make treatment of complex conditions challenging in primary care) [33].

The strengths of our study include the use of a large nationwide primary care database that includes data recorded by general practitioners, which reduced recall bias. However, some limitations should be recognised. First, our results may overestimate actual BZD intake, as we used prescribing data rather than filled prescription or used medications. Second, BZD and Z‐drugs prescribed by specialists or other health professionals are not accounted for in the MedicineInsight database, which may underestimate the real prevalence [38]. Third, the dataset does not provide reliable information about the reason for these prescriptions (i.e., reason not recorded at all or recorded using free‐text instead of standard medical codes/terminology), referrals to specialised services, duration of use or any other criteria to assess the appropriateness of BZD and Z‐drugs prescriptions. All that information would have been helpful in identifying potential areas for targeted intervention (deprescribing or non‐pharmacological options depending on the health condition) in specific socioeconomic, age and gender groups. Finally, practices were recruited to MedicineInsight using non‐random sampling, and the sample is not representative of the Australian population [22]. Therefore, although practices in MedicineInsight represent 8.2% of all Australian practices, differences between regions (i.e., remoteness and IRSAD, which are based on postcodes) should be interpreted with caution.

## 5 CONCLUSION

Although the prescription of most BZD and Z‐drugs declined over time, short‐intermediate‐acting BZD prescriptions remained higher among older women and long‐acting BZD were more frequent among younger men, especially if they were living in more disadvantaged areas. These groups would benefit from targeted interventions aiming to reduce the use of these medications and provide better access to non‐pharmacological treatment options, so that the burden of BZD‐related adverse health outcomes can be minimised. Moreover, these targeted strategies need to take into account the specific needs and responses in these two groups [28]. For older women, addressing system barriers to support non‐pharmacological therapies and minimise BZD dependency is a priority. For younger males, the higher use of long‐acting BZD may reflect gender differences in help‐seeking behaviour, and interventions should offer alternatives that consider social, occupational and lifestyle patterns [28, 36, 37].

## AUTHOR CONTRIBUTIONS

Each author certifies that their contribution to this work meets the standards of the International Committee of Medical Journal Editors.

## CONFLICT OF INTEREST

None to declare. No external funding was received by any authors for this study.

## Supporting information


**Table S1.** Comparison of all patients in MedicineInsight with those included in the study according to sociodemographic characteristics and prescription of benzodiazepines and Z‐drugs. Patients aged 18+ years.

**Table S2:** Prescription of very short‐acting benzodiazepines per 1000 consultations by sociodemographic characteristics and average percent annual change from 2011 to 2018—crude results.

**Table S3.** Prescription of very short‐acting benzodiazepines per 1000 consultations by sociodemographic characteristics and average percent annual change from 2011 to 2018—adjusted results.

**Table S4.** Prescription of short‐intermediate‐acting benzodiazepines per 1000 consultations by sociodemographic characteristics and average percent annual change from 2011 to 2018—crude results.

**Table S5.** Prescription of short‐intermediate‐acting benzodiazepines per 1000 consultations by sociodemographic characteristics and average percent annual change from 2011 to 2018—djusted results.

**Table S6.** Prescription of long‐acting benzodiazepines per 1000 consultations by sociodemographic characteristics and average percent annual change from 2011 to 2018—crude results.

**Table S7.** Prescription of long‐acting benzodiazepines per 1000 consultations by sociodemographic characteristics and average percent annual change from 2011 to 2018—adjusted results.

**Table S8.** Prescription of Z‐drugs per 1000 consultations by sociodemographic characteristics and average percent annual change from 2011 to 2018—crude results.

**Table S9.** Prescription of Z‐drugs per 1000 consultations by sociodemographic characteristics and average percent annual change from 2011 to 2018—adjusted results.

**Table S10.** Proportion of patients prescribed very short‐acting benzodiazepines by sociodemographic characteristics and average percent annual change from 2011 to 2018—adjusted results.

**Table S11.** Proportion of patients prescribed short‐intermediate‐acting benzodiazepines by sociodemographic characteristics and average percent annual change from 2011 to 2018—adjusted results.

**Table S12.** Proportion of patients prescribed long‐acting benzodiazepines by sociodemographic characteristics and average percent annual change from 2011 to 2018—adjusted results.

**Table S13.** Proportion of patients prescribed Z‐drugs by sociodemographic characteristics and average percent annual change from 2011 to 2018 – Adjusted results.

## References

[1] The Royal Australian College of General Practitioners . Prescribing drugs of dependence in general practice, Part B—Benzodiazepines. Melbourne: The Royal Australian College of General Practitioners, 2015. 100 Wellington Parade East Melbourne Victoria 3002 Australia. [cited 2021 Mar 13]. Available from: https://www.racgp.org.au/clinical‐resources/clinical‐guidelines/key‐racgp‐guidelines/view‐all‐racgp‐guidelines/drugs‐of‐dependence/part‐b.

[2] Poly TN , Islam MM , Yang H‐C , Li Y‐C . Association between benzodiazepines use and risk of hip fracture in the elderly people: a meta‐analysis of observational studies. Joint Bone Spine. 2020;87:241–9. 31778821 10.1016/j.jbspin.2019.11.003

[3] Lucchetta RC , da Mata BPM , Mastroianni PC . Association between development of dementia and use of benzodiazepines: a systematic review and meta‐analysis. Pharmacotherapy. 2018;38:1010–20. 30098211 10.1002/phar.2170

[4] Australian Institute of Health and Welfare (AIHW) . Alcohol, tobacco & other drugs in Australia. AIHW 2020 [cited 2021 Apr 16]. Available from: https://www.aihw.gov.au/reports/phe/221/alcohol‐tobacco‐other‐drugs‐australia/contents/drug‐types/non‐medical‐use‐of‐pharmaceutical‐drugs.

[5] Torres‐Bondia F , de Batlle J , Galván L , Buti M , Barbé F , Piñol‐Ripoll G . Trends in the consumption rates of benzodiazepines and benzodiazepine‐related drugs in the health region of Lleida from 2002 to 2015. BMC Public Health. 2020;20:818. 32487058 10.1186/s12889-020-08984-z PMC7268471

[6] Olfson M , King M , Schoenbaum M . Benzodiazepine use in the United States. JAMA Psychiat. 2015;72:136–42. 10.1001/jamapsychiatry.2014.1763 25517224

[7] Jackson G , Gerard C , Minko N , Parsotam N . Variation in benzodiazepine and antipsychotic use in people aged 65 years and over in New Zealand. N Z Med J. 2014;127:67–78. 24997465

[8] Harrison SL , Sluggett JK , Lang C , Whitehead C , Crotty M , Corlis M , et al. The dispensing of psychotropic medicines to older people before and after they enter residential aged care. Med J Aust. 2020;212:309–13. 32045014 10.5694/mja2.50501

[9] Zheng D , Brett J , Daniels B , Buckley NA , Pearson SA , Schaffer AL . Potentially inappropriate benzodiazepine use in Australian adults: a population‐based study (2014–2017). Drug Alcohol Rev. 2020;39:575–82. 32391624 10.1111/dar.13086

[10] Weymann D , Gladstone EJ , Smolina K , Morgan SG . Long‐term sedative use among community‐dwelling adults: a population‐based analysis. CMAJ Open. 2017;5:E52–60. 10.9778/cmajo.20160056 PMC5378535 28401119

[11] Miller CB , Valenti L , Harrison CM , Bartlett DJ , Glozier N , Cross NE , et al. Time trends in the family physician management of insomnia: the Australian experience (2000‐2015). J Clin Sleep Med. 2017;13:785–90. 28454597 10.5664/jcsm.6616 PMC5443739

[12] Agarwal SD , Landon BE . Patterns in outpatient benzodiazepine prescribing in the United States. JAMA Netw Open. 2019;2:e187399. 30681713 10.1001/jamanetworkopen.2018.7399 PMC6484578

[13] Brett J , Murnion B . Management of benzodiazepine misuse and dependence. Aust Prescr. 2015;38:152–5. 26648651 10.18773/austprescr.2015.055 PMC4657308

[14] Australian Government Department of Health . The Pharmaceutical Benefits Scheme (PBS). 2020 [cited 2020 May 28]. Available from: https://www.pbs.gov.au/pbs/home.

[15] Préville M , Bossé C , Vasiliadis H‐M , Voyer P , Laurier C , Berbiche D , et al. Correlates of potentially inappropriate prescriptions of benzodiazepines among older qdults: results from the ESA study. Can J Aging. 2012;31:313–22. 22800936 10.1017/S0714980812000232

[16] Yu N‐W , Chen P‐J , Tsai H‐J , Huang C‐W , Chiu Y‐W , Tsay W‐I , et al. Association of benzodiazepine and Z‐drug use with the risk of hospitalisation for fall‐related injuries among older people: a nationwide nested case–control study in Taiwan. BMC Geriatr. 2017;17:140. 28693443 10.1186/s12877-017-0530-4 PMC5504671

[17] Schifano F , Chiappini S , Corkery JM , Guirguis A . An insight into Z‐drug abuse and dependence: an examination of reports to the European medicines agency database of suspected adverse drug reactions. Int J Neuropsychopharmacol. 2019;22:270–7. 30722037 10.1093/ijnp/pyz007 PMC6441128

[18] Baldwin DS , Aitchison K , Bateson A , Curran HV , Davies S , Leonard B , et al. Benzodiazepines: Risks and benefits. A reconsideration. J Psychopharmacol. 2013;27:967–71. 24067791 10.1177/0269881113503509

[19] Cadogan CA , Ryan C , Cahir C , Bradley CP , Bennett K . Benzodiazepine and Z‐drug prescribing in Ireland: analysis of national prescribing trends from 2005 to 2015. Br J Clin Pharmacol. 2018;84:1354–63. 29488252 10.1111/bcp.13570 PMC5980334

[20] Smith AJ , Tett SE . How do different age groups use benzodiazepines and antidepressants? Drugs Aging. 2009;26:113–22. 19220068 10.2165/0002512-200926020-00003

[21] Chen L , Bell JS , Visvanathan R , Hilmer SN , Emery T , Robson L , et al. The association between benzodiazepine use and sleep quality in residential aged care facilities: a cross‐sectional study. BMC Geriatr. 2016;16:196. 27888835 10.1186/s12877-016-0363-6 PMC5124287

[22] Busingye D , Gianacas C , Pollack A , Chidwick K , Merrifield A , Norman S , et al. Data resource profile: MedicineInsight, an Australian national primary health care database. Int J Epidemiol. 2019;48:1741–1741h. 31292616 10.1093/ije/dyz147

[23] Benchimol EI , Smeeth L , Guttmann A , Harron K , Moher D , Petersen I , et al. The REporting of studies conducted using observational routinely‐collected health data (RECORD) statement. PLoS Med. 2015;12:e1001885. 26440803 10.1371/journal.pmed.1001885 PMC4595218

[24] Woods A , Begum M , Gonzalez‐Chica D , Bernardo C , Hoon E , Stocks N . Long‐term benzodiazepines and z‐drug prescribing in Australian general practice between 2011 and 2018: a national study. Pharmacol Res Perspect. 2022;10:e00896. 34918876 10.1002/prp2.896 PMC8929365

[25] Australian Bureau of Statistics , 2018. Australian Socio‐Economic Indexes for Areas (SEIFA). ABS Catalogue No 2033.0.55.001.

[26] Kaufmann CN , Spira AP , Depp CA , Mojtabai R . Long‐term use of benzodiazepines and nonbenzodiazepine hypnotics, 1999‐2014. Psychiatr Serv. 2018;69:235–8. 29089011 10.1176/appi.ps.201700095 PMC5794624

[27] Eriksen SI , Bjerrum L . Reducing prescriptions of long‐acting benzodiazepine drugs in Denmark: a descriptive analysis of nationwide prescriptions during a 10‐year period. Basic Clin Pharmacol Toxicol. 2015;116:499–502. 25382355 10.1111/bcpt.12347

[28] Liddon L , Kingerlee R , Barry JA . Gender differences in preferences for psychological treatment, coping strategies, and triggers to help‐seeking. Br J Clin Psychol. 2018;57:42–58. 28691375 10.1111/bjc.12147

[29] Penington Institute . Australia's annual overdose report 2019. ISBN: 978‐0‐9808778‐4‐7. Melbourne: Penington Institute; 2019 [cited 2021 Apr 3]. Available from: https://www.penington.org.au/australias-annual-overdose-report-2019-released/

[30] Airagnes G , Lemogne C , Renuy A , Goldberg M , Hoertel N , Roquelaure Y , et al. Prevalence of prescribed benzodiazepine long‐term use in the French general population according to sociodemographic and clinical factors: findings from the CONSTANCES cohort. BMC Public Health. 2019;19:566. 31088561 10.1186/s12889-019-6933-8 PMC6518636

[31] Autralian Bureau of Statistics . Mental Health. Health Conditions and Risk 2017–2018. 2021 [cited 2021 Aug 19]. Available from: https://www.abs.gov.au/statistics/health/health-conditions-and-risks/mental-health/2017-18.

[32] Macintyre A , Ferris D , Gonçalves B , Quinn N . What has economics got to do with it? The impact of socioeconomic factors on mental health and the case for collective action. Palgrave Communications. 2018;4:10.

[33] Haycock J , Grivell N , Redman A , Saini B , Vakulin A , Lack L , et al. Primary care management of chronic insomnia: a qualitative analysis of the attitudes and experiences of Australian general practitioners. BMC Fam Pract. 2021;22:158. 34294049 10.1186/s12875-021-01510-z PMC8299615

[34] Giebel C , Corcoran R , Goodall M , Campbell N , Gabbay M , Daras K , et al. Do people living in disadvantaged circumstances receive different mental health treatments than those from less disadvantaged backgrounds? BMC Public Health. 2020;20:651. 32393305 10.1186/s12889-020-08820-4 PMC7216680

[35] Begum M , Gonzalez‐Chica D , Bernardo C , Woods A , Stocks N . Trends in the prescription of drugs used for insomnia in Australian general practice, 2011‐2018. Br J Gen Pract. 2021;71:e877–86. 33950853 10.3399/BJGP.2021.0054 PMC8366783

[36] Sweetman A , Putland S , Lack L , McEvoy RD , Adams R , Grunstein R , et al. The effect of cognitive behavioural therapy for insomnia on sedative‐hypnotic use: a narrative review. Sleep Med Rev. 2020;56:101404. 33370637 10.1016/j.smrv.2020.101404

[37] Sweetman A , Knieriemen A , Hoon E , Frank O , Stocks N , Natsky A , et al. Implementation of a digital cognitive behavioral therapy for insomnia pathway in primary care. Contemp Clin Trials. 2021;107:106484. 34129952 10.1016/j.cct.2021.106484

[38] Delcher C , Harris DR , Park C , Strickler GK , Talbert J , Freeman PR . “doctor and pharmacy shopping”: a fading signal for prescription opioid use monitoring? Drug Alcohol Depend. 2021;221:108618. 33677354 10.1016/j.drugalcdep.2021.108618 PMC8026641

