# Supplementary material for: Trends and patterns of benzodiazepines and Z‐drugs prescriptions in Australian general practice: A national study (2011–2018)
Source: Drug Alcohol Rev. 2022 Oct 10;42(2):427–38. doi: 10.1111/dar.13561 (PMC10092554; doi:10.1111/dar.13561)
Supplement: Supplementary file 1 — Table S1. Comparison of all patients in MedicineInsight with those included in the study according to sociodemographic characteristics and prescription of benzodiazepines and Z‐drugs. Patients aged 18+ years. [file DAR-42-427-s011.docx]

Table S1. Comparison of all patients in MedicineInsight with those included in the study^a^ according to sociodemographic characteristics and prescription of benzodiazepines and Z-drugs. Patients aged 18+ years

|  | **All patients in MedicineInsight** | **Patients included in the study^a^** |
| --- | --- | --- |
|  | **n=2,356,616**  **%** | **n=1,450,613**  **%** |
| Practice remoteness |  |  |
| Major cities | 68.1 | 63.7 |
| Inner regional | 20.3 | 22.5 |
| Outer/remote/very remote | 11.6 | 13.7 |
| Practice IRSAD |  |  |
| Upper quintile (most advantaged) | 26.0 | 26.7 |
| 2nd upper quintile | 21.3 | 16.9 |
| Intermediate quintile | 24.2 | 22.7 |
| 2nd lower quintile | 14.4 | 14.9 |
| Lower quintile (most disadvantaged) | 14.2 | 18.8 |
| Patient age, years |  |  |
| 18-44 | 52.3 | 41.8 |
| 45-64 | 28.5 | 32.7 |
| ≥65 | 19.3 | 25.5 |
| Patient gender |  |  |
| Male | 43.4 | 42.0 |
| Female | 56.7 | 58.0 |
| Patient IRSAD |  |  |
| Upper quintile (most advantaged) | 25.8 | 24.8 |
| 2nd upper quintile | 20.8 | 17.2 |
| Intermediate quintile | 22.6 | 22.8 |
| 2nd lower quintile | 16.0 | 16.7 |
| Lower quintile (most disadvantaged) | 14.5 | 18.1 |
| Proportion of patients prescribed BZD or Z-drugs at any time between 2011-2018 |  |  |
| Very short-acting BZD | 0.09 | 0.18 |
| Intermediate-acting BZD | 6.0 | 12.0 |
| Long-acting BZD | 5.5 | 9.9 |
| Z-drugs | 1.2 | 2.1 |

^a^ Considering inclusion criteria for the practice (no gap of more than six weeks in data provision in the previous two years and a ratio between the highest and lowest number of annual consultations (2011-2018) lower than five) and the patients (at least three visits in every in any two consecutive years [e.g. a regular patient in 2018 had at least 3 clinical encounters from 1 January 2017 to 31 December 2018 for calendar year 2018], with at least one visit in each of these two consecutive years, aged 18+ years and attending general practices participating in MedicineInsight from 1 January 2011 to 31 December 2018). Due to the large sample size, all comparisons between patients included in the study vs. those not included returned *p*-values <0.01.

BZD, benzodiazepines; IRSAD, Index of Relative Socio-economic Advantage and Disadvantage.
