# Supplementary material for: Trends and patterns of benzodiazepines and Z‐drugs prescriptions in Australian general practice: A national study (2011–2018)
Source: Drug Alcohol Rev. 2022 Oct 10;42(2):427–38. doi: 10.1111/dar.13561 (PMC10092554; doi:10.1111/dar.13561)
Supplement: Supplementary file 11 — Table S11. Proportion of patients prescribed short‐intermediate‐acting benzodiazepines by sociodemographic characteristics and average percent annual change from 2011 to 2018—adjusted results. [file DAR-42-427-s008.docx]

Table S11. Proportion of patients prescribed short-intermediate-acting benzodiazepines by sociodemographic characteristics and average percent annual change from 2011-2018 – Adjusted results^a^

|  | **2011** | **2012** | **2013** | **2014** | **2015** | **2016** | **2017** | **2018** | **Average annual percent change (95% CI)^b^** | ***p*-value*** |
| --- | --- | --- | --- | --- | --- | --- | --- | --- | --- | --- |
|  | **n=615,366** | **n=676,151** | **n=742,979** | **n=815,797** | **n=892,744** | **n=975,266** | **n=1,064,820** | **n=1,184,993** |  |  |
| **Overall** | 7.2 | 7.3 | 7.2 | 6.8 | 6.4 | 6.2 | 6.0 | 5.0 | -4.9 (-5.4; -3.5) | <0.001 |
| Practice remoteness |  |  |  |  |  |  |  |  |  |  |
| Major cities | 7.2 | 7.3 | 7.2 | 6.8 | 6.4 | 6.2 | 5.9 | 5.0 | -5.0 (-5.7; -4.4) | <0.001 |
| Inner regional | 7.6 | 7.7 | 7.4 | 7.2 | 6.8 | 6.7 | 6.4 | 5.4 | -4.6 (-5.4; -3.8) | <0.001 |
| Outer/remote/very remote | 6.5 | 6.5 | 6.4 | 6.4 | 5.9 | 5.8 | 5.5 | 4.5 | -5.3 (-6.6; -4.1) | <0.001 |
| Practice IRSAD |  |  |  |  |  |  |  |  |  |  |
| Upper quintile (most advantaged) | 7.9 | 7.9 | 7.6 | 7.4 | 7.1 | 6.9 | 6.6 | 5.6 | -4.7 (-5.3-4.0) | <0.001 |
| 2nd upper quintile | 7.4 | 7.5 | 7.3 | 7.1 | 6.5 | 6.3 | 6.2 | 5.3 | -4.8 (-5.7; -3.9) | <0.001 |
| Intermediate quintile | 6.8 | 6.9 | 6.8 | 6.5 | 6.2 | 6.1 | 5.8 | 5.0 | -4.1 (-5.0; -3.2) | <0.001 |
| 2nd lower quintile | 7.0 | 7.4 | 7.4 | 6.7 | 6.3 | 6.0 | 5.5 | 4.6 | -6.1 (-8.0; -4.1) | <0.001 |
| Lower quintile (most disadvantaged) | 6.9 | 6.8 | 6.6 | 6.4 | 5.9 | 5.7 | 5.4 | 4.3 | -5.7 (-6.8-4.7) | <0.001 |
| Patient age, years |  |  |  |  |  |  |  |  |  |  |
| 18-44 | **3.8** | **3.9** | **3.7** | **3.4** | **3.1** | **3.0** | **2.8** | **2.3** | -7.1 (-7.8; -6.4) | <0.001 |
| 45-64 | **7.2** | **7.1** | **6.9** | **6.5** | **6.1** | **5.9** | **5.5** | **4.8** | -5.5 (-6.0; -5.0) | <0.001 |
| ≥65 | **12.0** | **11.9** | **11.8** | **11.3** | **10.8** | **10.6** | **10.4** | **9.1** | -3.6 (-4.0; -3.1) | <0.001 |
| Patient gender |  |  |  |  |  |  |  |  |  |  |
| Male | **5.3** | **5.5** | **5.4** | **5.1** | **4.9** | **4.8** | **4.6** | **3.9** | -4.2 (-4.8; -3.6) | <0.001 |
| Female | **8.6** | **8.6** | **8.5** | **8.1** | **7.6** | **7.3** | **7.0** | **5.9** | -5.2 (-5.6; -4.8) | <0.001 |
| Patient IRSAD |  |  |  |  |  |  |  |  |  |  |
| Upper quintile (most advantaged) | **7.1** | **7.1** | **7.0** | **6.8** | **6.4** | **6.1** | 5.9 | 5.0 | -4.4 (-5.0; -3.8) | <0.001 |
| 2nd upper quintile | **6.9** | **6.9** | **6.8** | **6.6** | **6.2** | **5.9** | 5.8 | 4.9 | -4.6 (-5.1; -4.0) | <0.001 |
| Intermediate quintile | **7.2** | **7.3** | **7.1** | **6.8** | **6.4** | **6.4** | 6.0 | 5.0 | -4.4 (-5.0; -3.8) | <0.001 |
| 2nd lower quintile | **7.2** | **7.4** | **7.2** | **6.8** | **6.5** | **6.2** | 5.9 | 4.9 | -5.9 (-7.4; -4.3) | <0.001 |
| Lower quintile (most disadvantaged) | **8.0** | **8.0** | **7.9** | **7.2** | **6.7** | **6.7** | 6.4 | 5.4 | -5.7 (-6.5; -4.9) | <0.001 |

^a^ Marginal adjusted frequencies (per 1,000 consultations) based on logistic regression models. Practice remoteness and practice IRSAD were mutually adjusted (Model 1). Patient age, gender and IRSAD were mutually adjusted and also for practice remoteness and practice IRSAD (Model 2).

^b^ Average percent annual change based on adjusted Poisson regression models with the additional inclusion of multiplicative interaction terms between each covariate and the year (as an ordinal variable). Rate ratios obtained from the Poisson regression models were then converted to a percentage (i.e. (rate ratio – 1) × 100).

* *p*-value for trend for the average percent annual change in prescription rates between 2011-2018 in the same category.

CI, confidence interval; IRSAD, Index of Relative Socio-economic Advantage and Disadvantage.
